# Supplementary material for: A vaccine central in A(H5) influenza antigenic space confers broad immunity
Source: Nature. 2025 Oct 15;647(8091):1005–13. doi: 10.1038/s41586-025-09626-3 (PMC12657240; doi:10.1038/s41586-025-09626-3)
Supplement: Supplementary file 1 — Supplementary Notes 1–4, the legends for Supplementary Tables 1–11, the legends for Supplementary Data 1–10 and the legend for Supplementary Video 1. [file 41586_2025_9626_MOESM1_ESM.pdf]

nature portfolio

https://doi.org/10.1038/s41586-025-09626-3

Supplementary information

A vaccine central in A(H5) influenza antigenic space confers broad immunity

In the format provided by the authors and unedited

Supplementary information for
A vaccine central in A(H5) influenza antigenic space confers broad immunity

Adinda Kok[1]†, Samuel H. Wilks[2]‡§, Sina Tureli[2]§, Sarah L. James[2]§, Theo M. Bestebroer[1], David F. Burke[2]¶, Mathis Funk[1]#, Stefan van der Vliet[1], Monique I. Spronken[1], Willemijn F. Rijnink[1], David Pattinson**, Dennis de Meulder[1], Miruna E. Rosu[1], Pascal Lexmond[1], Judith M.A. van den Brand[3], Sander Herfst[1], Derek J. Smith[2], Ron A.M. Fouchier[1], Mathilde Richard[1]*

Affiliations

[1] Department of Viroscience, Erasmus University Medical Center; Rotterdam, The Netherlands.

[2] Center for Pathogen Evolution, University of Cambridge; Cambridge, United Kingdom.

[3] Division of Pathology, Faculty of Veterinary Medicine, Utrecht University; Utrecht, the Netherlands

* Email: m.richard@erasmusmc.nl

† Present address: Biomolecular Mass Spectrometry and Proteomics, Bijvoet Center for Biomolecular Research, Department of Chemistry, Faculty of Science, Utrecht University; Utrecht, The Netherlands.

‡ Present address: Institute for Electrical and Electronic Engineering, Faculty of Engineering and Information Technology, University of Melbourne; Melbourne, Australia

§ These authors contributed equally to this work

¶ Present address: Centre for Host-Microbiome Interactions, King's College, London Tower Wing, Guy's Hospital; London, United Kingdom.

Deceased

** Present address: Influenza Research Institute, Department of Pathobiological Sciences, School of Veterinary Medicine, University of Wisconsin-Madison; Madison, USA

Table of content

Supplementary Notes **3**

Supplementary Note 1 3
Supplementary Note 2 4
Supplementary Note 3 7
Supplementary Note 4 10

Supplementary Tables Legends **12**

Supplementary Table 1 12
Supplementary Table 2 12
Supplementary Table 3 12
Supplementary Table 4 13
Supplementary Table 5 13
Supplementary Table 6 13
Supplementary Table 7 13
Supplementary Table 8 13
Supplementary Table 9 13
Supplementary Table 10 14
Supplementary Table 11 14

Supplementary Data Legends **15**

Supplementary Data 1 15
Supplementary Data 2 15
Supplementary Data 3 15
Supplementary Data 4 16
Supplementary Data 5 16
Supplementary Data 6 16
Supplementary Data 7 17
Supplementary Data 8 17
Supplementary Data 9 17
Supplementary Data 10 17

Supplementary Video Legend **18**

Supplementary Notes

Supplementary Note 1 | Compilation of the hemagglutination inhibition (HI) dataset for the A(H5) antigenic map.

The A(H5) map dataset was generated using HI data from multiple individual assays. The 127 antigens titrated against 33 post-infection sera resulted in a total of 4191 datapoints. Each data point, i.e. each combination of antigen and serum, was titrated 1.7 times on average, and 48% of antigen-serum pairs were titrated two or more times. The variability between assays was evaluated for the antigen-serum combinations which were assessed in multiple independent HI assays. The standard deviation (SD) of $\log_2$ transformed HI titres between independent assays was below 1.5 for 93% of the data points which were titrated two or more times, with an average of 0.46 (Extended Data Fig. 1a). Of note, one antigenic unit (AU) difference corresponds to an SD of 0.7, meaning that differences of one or two AUs represent most of the observed HI assay variation. The HI data of individual assays were compiled into a merged dataset through the 'mergeMaps' function in Racmacs, using the 'table' method and the merge options settings of method = 'conservative' and sd_limit = 1.5. A sample SD of 1.5 allows for the merging of titres within a four-fold difference, which is generally considered as the maximum acceptable variation between HI assays. Titres which were outside of the assay's detection limit, denoted with a 'smaller-than' or 'larger-than' sign, were converted to the closest numerical titre (e.g., a <10 is converted to a 5) for calculation of the sample SD. Generally, for each antigen-serum combination, the resulting merged titre was the geometric mean of all measured values. In the case of threshold titres, if the SD was below the set limit of 1.5, the merged titre was the highest 'smaller-than' or the lowest 'larger-than' which satisfied all the measured values. For 145 antigen and serum combinations (3.46% of the 4191 antigen and serum combinations in the full dataset), the sample SD of the titration repeats was above 1.5, and consequently the titre was set to 'NA' and considered as unknown for the generation of the antigenic map. The resulting merged dataset was used as a basis for the generation of the antigenic map (Supplementary Table 4).

Supplementary Note 2 | A(H5) antigenic map dimensionality.

Firstly, we investigated the number of dimensions required to best represent the A(H5) HI data in an antigenic map. Generally, for a map of *n* dimensions, each point should have a minimum of *n* detectable titres (i.e., non-threshold and non-'NA' titres) to be placed in the antigenic map, and a minimum of *n*+1 detectable titres for accurate placement. In the cross-HI dataset, 13 antigens had less than five detectable titres (Supplementary Table 4). To allow for a fair comparison between dimensions one to five, we excluded these antigens and their five corresponding homologous sera, resulting in a dataset with 114 antigens and 28 sera (Supplementary Table 2).

A dimension test was performed using the 'dimensionTestMap' function in Racmacs. For dimensions one to five, 100 antigenic maps were generated from 100 random start positions, each of them performed with 1000 optimizations. In each of these 100 repeats, 10% of the data were randomly excluded, and subsequently predicted from the resulting map. For each repeat, the root mean square error (RMSE) between predicted and measured titres was calculated. A comparison of the mean RMSE in each dimension is indicative of the dimension required to represent the data well (Extended Data Fig. 1b).

The largest improvement in mean RMSE was observed between dimensions one and two (mean RMSE difference of 0.44), followed by dimensions two and three (mean RMSE difference of 0.28), suggesting that three dimensions represent the HI data substantially better (Extended Data Fig. 1b). A slight improvement was observed between three and four dimensions (mean RMSE difference of 0.12), and barely any improvement between four and five dimensions (mean RMSE difference of 0.02), indicating that more than four dimensions did not substantially improve representation of the data (Extended Data Fig. 1b). A comparable pattern was observed when comparing the correlation between pairwise antigen-serum HI table distances and pairwise antigen-serum Euclidian distances in the antigenic map across dimension one to five. The correlation improved only slightly between three and four dimensions (R^2 of 0.66 to 0.71), as compared to between one, two and three dimensions (R^2 of 0.24, 0.55 and 0.66, respectively) (Extended Data Fig. 1c). Similarly, the reduction in the total map stress, corresponding to the sum of the squared differences between table and map distances, was only minimal between three and four dimensions (3860 to 3160), as compared to that between one, two and three dimensions (12247, 5950 and 3860, respectively) (Extended Data Fig. 1d).

We further investigated whether the A(H5) cross-HI data should be represented in three or four dimensions. The differences in point positions between two maps were compared using a Procrustes analysis through the 'procrustesData' function in Racmacs. Given two antigenic maps with identical antigens, sera and dimensions, the Procrustes function aims to find the optimal Euclidean transformation

(translation, rotation, scaling, reflection) of the first map such that the distance between the coordinates of the two maps (called the Procrustes error) is minimized, i.e., the best superimposition is determined. The resulting difference in map coordinates for each point is the Procrustes distance, expressed in AU. This analysis can be visualized within the antigenic map (with the 'procrustesMap' function in Racmacs), where the point positions of the first map are visualized as usual, and arrows point to the position of the corresponding points in the comparison map. The length of an arrow corresponds to the Procrustes distance between the positions of a point in the two maps. Total Procrustes distances between two maps can then be expressed by calculating the root mean square (RMS) or the median Procrustes distance. When dimensions are not identical, one can supplement the lower dimensional map with extra coordinates of zero. The resulting map comparison indicates how the extra dimension is utilized to better fit the observed titres in the larger dimensional map, or, how the larger dimensional map is projected on to the lower dimensional map. Little difference was observed in the point positions between the three- and four-dimensional maps (RMS of 2.39 AU and median Procrustes distance of 1.49 AU). Moreover, the distances between antigens and sera in the map, and the individual antigen stresses correlated well between the three- and four-dimensional maps (Extended Data Fig. 1e and f, R^2 of 0.93 and 0.84, respectively).

To further compare the three- and four-dimensional maps, a piecewise Procrustes analysis was performed. To this end, we used a modified version of the Procrustes analysis to compare maps of different dimensions, which is available in PyRacmacs (https://github.com/iAvicenna/PyRacmacs). Code written for this analysis is available on Github (https://github.com/epiv-lab/H5-antigenic-evolution) and archived on Zenodo (https://doi.org/10.5281/zenodo.13237524). Given two maps and *n* the number of pieces, piecewise Procrustes aims to find the optimal partition of the coordinates into *n* pieces such that the sum of Procrustes errors is minimized. In essence, the different pieces of the map are allowed to move freely relative to each other when transitioning between dimensions. If one can obtain a lower Procrustes error between a lower and higher dimensional map when using few pieces, the lower dimensional map can be embedded in the higher dimensions by a relatively simple transformation such that it would look like the higher dimensional map, i.e. the two maps are geometrically similar. Here, we have used this method to compare the antigenic map in three and four dimensions. One can see that the three-dimensional map fits the four-dimensional map quite well when split into only two pieces (Extended Data Fig. 2, Supplementary Data 3a, b). These analyses suggest that the general topology of the map in four-dimensions is similar to that of the three-dimensional map.

Given that the three-dimensional map recapitulated the geometry of the four-dimensional map well, and that only minimal improvements in prediction error, correlation between HI table and antigenic map distances and overall stress were observed upon the use of the fourth dimension, we concluded that its use

to visualize A(H5) cross-HI data was not warranted. However, it is not excluded that four dimensions will be required with further expansion of the A(H5) map including novel antigens and sera.

Supplementary Note 3 | Analysis of three-dimensional A(H5) antigenic map.

The dataset initially contained 10 antigens that reacted with fewer than four detectable titres to ferret sera, insufficient to confidently place points in a map of three or more dimensions. This was despite the presence of homologous sera for four of them (A/chicken/West-Java/119/2010 (clade 2.1.3.2a), A/duck/Jiangxi/0114-NCJD064-P/2015 (clade 2.3.4.4f), A/Guandong/18SF020/2018, (clade 2.3.4.4h), A/chicken/Chiping/0321/2014 (clade 7.2)), which were raised in an attempt to better characterize low reactive antigens. Homologous titres against these ferret sera were high, ranging between 392 and 1580, indicating that these four antigens have divergent antigenic properties, rather than being generally low reactive in HI assays. However, the possibility of the other six viruses being low reactive rather than antigenically divergent cannot be excluded without the presence of a homologous serum. To allow placement in the three-dimensional antigenic map, these antigens and, if available, corresponding homologous sera, were removed from the dataset (Supplementary Tables 2 and 3), but raw HI reactivity patterns are shown in Supplementary Table 4. The final dataset used to generate the A(H5) three-dimensional map contained 117 antigens and 29 sera (117x29, Supplementary Table 2). In this dataset, 127 out of the 3393 (3.74%) titres were set to 'NA' due to the SD of $\log_2$ transformed HI titres of repeats being above 1.5. The resulting map geometry was close to identical to the three-dimensional map used in the dimension investigation above (Procrustes RMS of 0.67 AU, median Procrustes difference of 0.31 AU). The correlation between the distances obtained from the HI table and from the map was comparable to that previously observed in the dimension testing described above ($R^2 = 0.64$) (Extended Data Fig. 3a). The point positions were further validated using the 'moveTrappedPoints' and 'checkHemisphering' functions (Racmacs). Here, each point is moved individually in the map to assess whether better (trapped points) or equal (hemisphering points) local optima, i.e. positions with a lower or equal point stress as compared to its original position respectively, are found upon relaxing of the map (optimization of point positions starting from their current coordinates). These analyses revealed that no points located in local optima, nor hemisphering points, were detected in this map.

We then investigated whether better maps, i.e. with lower total stress, could be generated using dimensional annealing, through the 'make.acmap' function (Racmacs) with the option of dim_annealing set to 'TRUE'. Using this method, each optimization of the map is first optimized in five dimensions. The dimension is then reduced by one, the map is relaxed and optimized again, until the set dimension is reached. The three-dimensional map generated using dimensional annealing had a higher total stress (4158) as compared to the map generated using the default settings (4136), indicating that dimensional annealing did not allow the generation of a better antigenic map.

The stability of the map was first assessed by investigating the effect of removing each single individual antigen and serum. The median Procrustes distance of all points was determined for each resulting map, and the distribution was plotted as histograms (Extended Data Fig. 3b, c). Generally, only minimal changes in the map positions were observed upon removing individual antigens or sera, with a mean median difference of 0.09 AU and 0.34 AU when removing individual antigens and sera, respectively. Removal of three individual antigens and five individual sera led to maps with median Procrustes distances above 0.40 AU and 0.60 AU, respectively (Extended Data Fig. 3b, c). These maps were compared to the full map in further detail (Supplementary Data 4). In six out of eight maps, the difference was mainly the result of significant changes in the positions of clade 2.3.4.4 antigens and sera. In addition, large changes in positions were observed for several antigens located on the periphery of the antigenic map. Most notably, the position of the A/turkey/Wisconsin /1968 antigen (non-GsGd) changed substantially in four out of the eight maps. Taken together, this analysis indicated that the three-dimensional map geometry was generally robust and insensitive to the absence of individual antigens and sera.

To assess the certainty in point positions in the antigenic map, we used two tests. Firstly, triangulation blobs, indicating the area in which a particular point can be located without increasing the total map stress by more than one unit, were generated with the 'triangulationBlobs' function (Racmacs) with the option of 'grid_spacing' = 0.25. This analysis revealed that, while the overall geometry of the map was consistent, the positions of points at the periphery of the map were less well coordinated than those located in the centre, which is expected given that peripheral points show overall lower HI reactivity and are not constrained by surrounding sera (Supplementary Data 3c). Secondly, Bayesian bootstrapping was performed through the 'bootstrapMap' function in Racmacs for 1000 repeats with 100 optimizations per repeat, to understand the confidence of the positions of each point in the map. For each repeat, weights were randomly assigned to each titre in the HI data used to construct the map. For each point, the blob which encompassed the positions of 680 of the 1000 bootstrap runs (corresponding to a SD of 1) was computed using the 'bootstrapBlobs' function in Racmacs. The volumes of the resulting bootstrap blobs were calculated with a functionality available in PyRacmacs (https://github.com/iAvicenna/PyRacmacs) by fitting a triangular mesh to each bootstrap point using the package trimesh (version 3.2.0, https://trimsh.org/). Code to calculate bootstrap volumes and colour-code the map accordingly is available on Github (https://github.com/epiv-lab/H5-antigenic-evolution) and archived on Zenodo (https://doi.org/10.5281/zenodo.13237524). The radius of a sphere of equal volume, expressed in AU, was used as an indicator of the uncertainty of point position (Extended Data Fig. 3e, g). Generally, points which are well triangulated have spherical blobs, and thus the reported radius gives a good estimate of their uncertainty. A good correspondence between blob maximum width and sphere radius was observed for most of the points, indicating that most blobs were indeed spherical (Extended Data Fig. 3f, h). Bootstrap blob

volumes were generally larger for antigens located at the periphery of the antigenic map (Supplementary Data 3f), in accordance with the result of the triangulation blob analysis. Generally, the largest bootstrap blobs were observed for the non-GsGd and clade 1 antigens, suggesting a higher degree of uncertainty in their positioning in the map.

Further analysis on the map stability was performed by comparing its individual optimizations. Upon generation of the antigenic map, 1000 optimizations were performed, resulting in 1000 maps which were subsequently sorted by ascending stress, i.e., optimization (opt.) 1 corresponds to the one with lowest stress. The positions of points and the total map stress of the best map (opt. 1) were compared to those from subsequent optimizations (Extended Data Fig. 4a, b, respectively). Conformations of the 567 best optimizations were generally similar to opt. 1, and differences in point positions indicated by the median Procrustes distance (Extended Data Fig. 4a) were the result of relatively big changes in position of only a few points. Interestingly, from optimization 568 onwards, a total of 138 maps with similar Procrustes distance distributions and total map stress were observed (Extended Data Fig. 4). Further investigation of these maps revealed that their conformations were virtually identical to one another, but significantly different from opt. 1. This alternative map conformation (Supplementary Data 3d) represented the current data slightly less well than opt. 1 (R^2 of the linear regression of HI table versus map distances of 0.6322 for opt. 568 as compared to 0.6373 for opt. 1). The main differences were found in positions of antigens at the periphery of the antigenic map, including the non-GsGd, clade 1 and clade 2.3.4.4 antigens map (Supplementary Data 3e). Generally, collective changes in the positions of genetically similar antigens were observed, suggesting similar topology, especially for central points. This analysis showed that the map conformation was relatively stable, since most low stress maps resulted in similar conformations as the lowest stress map, albeit with the occasional movement of individual points. However, the observation of an alternative conformation with comparable total stress and HI table versus map distances suggested metastability of the antigenic map. Considering the relatively minor difference in total map stress (4136 for optimization 1 and 4262 for optimization 568), it should be noted that the addition of new data could potentially favour either of these conformations in the future.

Supplementary Note 4 | Histopathology and immunohistochemistry analyses in pre-clinical ferret studies.

In the pre-clinical ferret studies, tissues obtained 4 days post-inoculation (dpi) were used for histopathological and immunohistochemistry (IHC) analyses. Generally, lesions in the respiratory tract were detected in all animals, but the extent differed between experimental groups and challenges. This first paragraph will describe the observed respiratory tract lesions qualitatively, and the following paragraphs will describe the quantitative differences between animals from the different experimental groups.

In the lungs, the lesions were mostly associated with the bronchioles and bronchi and characterized by a mild to moderate thickening of the alveolar septa with infiltration of few neutrophils, lymphocytes, plasma cells as well as variable interstitial oedema and epithelial necrosis. The alveolar lumina contained variable amounts of oedema and increased numbers of alveolar macrophages and occasional neutrophils. The bronchioles and bronchi showed exocytosis of neutrophils and lymphocytes with epithelial hyperplasia and hypertrophy. There was perivascular and peribronchiolar/bronchial cuffing and oedema as well as multifocal moderate type II hyperplasia. There was multifocal bronchoadenitis and bronchus associated lymphoid tissue (BALT) hyperplasia. In the trachea, there was mild exocytosis and focal loss of ciliated cells with occasionally multifocal tracheal adenitis with necrosis and neutrophils. In the nose, the epithelium was flattened with loss of ciliated cells and severe inflammation with exocytosis of neutrophils, and in the alveolar lumina, of many neutrophils, macrophages and cellular debris. In the lamina propria, there were variable numbers of neutrophils, lymphocytes, plasma cells and less macrophages.

The histological parameters that were scored are summarized in Supplementary Table 10. In animals from the H5N1$_{Giza}$ challenge study, significant differences in scoring were only observed between the Mock$_{VACC}$ and vaccinated groups, and not between the vaccinated groups. A general pattern was observed where the median scoring for severity and extent of alveolitis, severity of bronchitis/bronchiolitis, peribronchial cuffing, alveolar oedema and haemorrhage was highest in animals from the Mock$_{VACC}$ group, lower in those from the Anhui$_{VACC}$ and AC-Anhui$_{VACC}$ groups, and even lower in those from the Giza$_{VACC}$ group (Supplementary Table 10, Extended Data Fig. 11). Influenza virus nucleoprotein (NP) expression was detected in the alveoli, bronchioles, bronchi and trachea of the Mock$_{VACC}$ animals, but absent in animals from the vaccinated groups. In the noses, NP antigen expression was detected in animals from all groups with few cells positive, with the highest score observed in the Mock$_{VACC}$ ($p<0.05$ as compared to Anhui $_{VACC}$ and AC-Anhui$_{VACC}$).

In animals from the H5N6$_{Sichuan}$ challenge study, differences in histopathological scorings between groups were generally not statistically significant. Generally speaking, the severity and extent of alveolitis,

severity of bronchitis/bronchiolitis, tracheitis, peribronchial cuffing, alveolar oedema and haemorrhage was highest in animals from the $Mock_{VACC}$ group, slightly less high in those from $Anhui_{VACC,}$ group, and lower in those from the AC-$Anhui_{VACC}$ and $Sichuan_{VACC}$ groups, with $Sichuan_{VACC}$ animals having slightly lower scores (Supplementary Table 10, Extended Data Fig. 11) . Interestingly, the sum of severity and extent of alveolitis was significantly lower compared to the $Mock_{VACC}$ group in animals from the AC-$Anhui_{VACC}$ and $Sichuan_{VACC}$ groups, but not the $Anhui_{VACC}$ group (Supplementary Table 10, Extended Data Fig. 11). Scoring of NP antigen expression in the alveoli, bronchioles, bronchi and trachea were highest in animals from the $Mock_{VACC}$ group, lower in those from the $Anhui_{VACC}$ group, minimal to absent in those from the AC-$Anhui_{VACC}$ and $Sichuan_{VACC}$ groups. In the noses, few cells were positive for NP antigen expression in all groups.

Supplementary Tables Legends

Supplementary Table 1 | Accession numbers and names of HA sequences for generation of the phylogenetic tree.

For sequences obtained from the GISAID database (i.e. accession numbers starting with 'EPI'), information on the contributors of the sequences is indicated.

Supplementary Table 2 | Antigens selected for antigenic characterization and sera production.

Under 'Antigen', the names of the antigens as used in this study (virus name followed by the respective genetic clade, with 'C' for non-GsGd antigens) are listed. In the column 'Homologous serum', the names of the sera used in this study are indicated on the same row as the homologous antigen. Under 'Accession number', the accession numbers in the BV-BRC or GISAID (starting with 'EPI') databases are listed. The column 'Present in dataset 114x28' indicates whether the antigen was present in the dimension testing dataset, as detailed in Supplementary Note 2 and 3. The column 'Present in dataset 117x29' indicates whether an antigen was present in the final dataset. The column 'Virus type' indicates whether a full virus or a recombinant virus with the respective HA (without the multibasic cleavage site (MBCS) if applicable) in the background of PR/8 or PR/8 HY virus was used. The column 'Origin of the HA gene/virus' indicates how the corresponding antigen was procured. The column 'CVV-(like)' indicates whether an antigen is a WHO candidate vaccine virus (CVV), or if it is the closest matching antigen in our dataset to a CVV. For all A(H5) WHO CVVs that were not present in our dataset, the genetically closest antigen in our dataset (full HA amino acid level excluding the signal peptide) was determined. If the difference between the CVV and the respective antigen was less than ten amino acids, these are labelled 'CVV-like' and the amino acid differences are indicated in the 'Notes' column. In 'Notes', further details are listed.

Supplementary Table 3 | Ferret sera used for antigenic characterization.

Under 'Virus', the names of the virus used to generate the respective serum are indicated. Under 'Genetic clade', the respective HA genetic clade of the antigen is indicated. 'Virus type' indicates whether a full virus or a recombinant virus with the respective HA (without MBCS) in the background of PR/8 or PR/8 HY virus was used. Under 'Boost', a '+' indicates the use of a subcutaneous boost, and a '-' indicates that no boost was performed. Under 'HA' and 'NA', the names of the respective gene segments used are listed, and under 'HA accession number' and 'NA accession number', the corresponding database accession numbers are listed. 'PR/8 backbone' indicates whether PR/8 or PR/8 HY was used.

Supplementary Table 4 | Merged HI titre dataset used for generation of the A(H5) antigenic map.
Rows correspond to antigens and columns to sera. A subset of titrations was replicated twice or more to evaluate assay variation (Extended Data Fig. 1a). Titres for which repeat titrations varied above the set limit (titres set to 'NA', see Supplementary Note 1) were excluded from the dataset and indicated with an Asterix (*). The column right of the antigen names and the row below the sera are colour-coded based on the corresponding genetic clade as in Fig. 1. HI titres are colour-coded based on titre height.

Supplementary Table 5 | Pairwise distances between antigens in the A(H5) antigenic map.
Distances were extracted from the antigenic map displayed in Fig. 1b and Supplementary Data 2 and are expressed in antigenic units (AU).

Supplementary Table 6 | Resialylated turkey red blood cell assay with viruses carrying wild-type and mutated HAs.
Data of individual assays are separated by thick horizontal lines. Undetectable titres (<0,5 hemagglutination units (HAU)/25 µL) are denoted by '-'. TRBCs: turkey red blood cells; VCNA: *Vibrio cholerae* neuraminidase; α2,3: α2,3-sialyltransferase; α2,6: α2,6-sialyltransferase.

Supplementary Table 7 | HI reactivity of viruses carrying wild-type and mutated HAs.
For viruses carrying wild-type HAs, merged data from the full H5 map dataset are shown. For the viruses carrying mutated HAs, data of a single HI assay are shown.

Supplementary Table 8 | HI dataset of vaccination sera.
The first 10 columns contain the data of vaccination sera generated with whole-inactivated vaccines. The subsequent columns contain the data of sera from the vaccination-challenge studies. For each experimental group, titres of the mean sera and individual animal sera are shown. Titres which were not measured are indicated with an Asterix (*). Data of a single HI assay are shown. The column right of the antigen names is colour-coded based on the corresponding genetic clade as in Fig. 1. HI titres are colour-coded based on titre height. GMT: Geometric mean titre.

Supplementary Table 9 | HI titre dataset of vaccination sera from the vaccination-challenge studies.
HI titres of sera obtained pre- and post- boost vaccination against antigens used in the vaccination-challenge studies. Data of a single HI assay are shown.

Supplementary Table 10 | Summary of histopathological and immunohistochemistry scoring in the vaccination-challenge studies.

The median is indicated followed by the range in brackets (n=6). The severity of alveolitis, bronchiolitis, bronchitis, bronchial adenitis, tracheitis and rhinitis was scored as follows: 0: no inflammatory cells; 1: few inflammatory cells; 2: moderate numbers of inflammatory cells; 3: many inflammatory cells. The extent of alveolitis was scored as follows: 0: 0%; 1: 1–25%; 2: 25–50%; 3: >50%. The presence of alveolar oedema, alveolar haemorrhage and type II pneumocyte hyperplasia was scored as follows: 0: no; 1: yes. The extent of peribronchial, peribronchiolar and perivascular infiltrates was scored as follows: 0: none; 1: one to two cells thick; 2: three to ten cells thick; 3: more than ten cells thick. For the lung, cumulative scores for each animal are the percentage of fields positive for influenza virus NP antigen based on 25 arbitrarily chosen, 20x objective, fields of lung parenchyma. For the bronchioles and bronchi in the lung, the main bronchus, trachea and nose, the percentage of positively staining epithelium was estimated for each slide and averaged per animal. Significant differences between groups were assessed with a Kruskal-Wallis test followed by a pairwise two-sided Dunn's test with Bonferroni correction for multiple comparisons. All pairwise comparisons were tested. Only statistically significant differences ($p<0.05$) are highlighted with a symbol followed by the corresponding p value. A '*' indicates a significant difference with the $Mock_{VACC}$ group and a '†' indicates a significant difference with the $Giza_{VACC}$ group. A '‡' indicates that for one animal, data were not present.

Supplementary Table 11 | Non-coding regions of all gene segments of viruses used for the vaccination-challenge studies.

Listed are the virus name, the respective gene segment, the 3' non-coding regions, including the start codon and the 5' non-coding regions, including the stop codon.

Supplementary Data Legends

Supplementary Data 1 | A(H5) HA maximum likelihood phylogenetic tree.

Zoomable pdf version of the tree displayed in Fig. 1a. Visualization as described for Fig. 1a and, in addition, the isolate names corresponding to the HA sequences are shown. Available via https://epiv-lab.github.io/H5-antigenically-central-vaccine/Supplementary_Data_1.pdf.

Supplementary Data 2 | Three-dimensional A(H5) influenza antigenic map.

An interactive version of the three-dimensional antigenic map constructed from the final 117x29 dataset, shown in Fig. 1b. Antigens are displayed as closed spheres and sera are displayed as open cubes. Antigens and sera are colour-coded based on the genetic HA clade, as indicated on the right-hand side of the figure. Antigens and sera names can be visualized by hovering over the points. Each direction (x, y, z) represents antigenic distance, and one square of the grid corresponds to one antigenic unit, which is defined as a two-fold difference in HI titre. The antigenic map can be rotated by clicking and dragging in the panel. On the top right are different functions to explore the map and a brief description of each function appears when hovering over. The total map stress, mean stress per titre and mean stress per detectable titre are indicated at the bottom left. Available via https://epiv-lab.github.io/H5-antigenically-central-vaccine/Supplementary_Data_2.html.

Supplementary Data 3 | Validation of the A(H5) antigenic map.

Interactive versions of the three-dimensional antigenic map (117x29), represented as described for Supplementary Data 2. **a-b,** Piecewise Procrustes analysis (see Extended Data Fig. 2 and detailed in Supplementary Note 3) comparing the antigenic maps in three and four dimensions. The results of analysis with one (**a**) and two (**b**) pieces are displayed. The antigen colour hue indicates which piece it belongs to, and the shading indicates the Procrustes distance according to the gradient displayed on the right, in antigenic units (AU). **c,** Triangulation blobs indicating the area in which each datapoint can be located in the antigenic map without increasing the total map stress by more than one unit. **d,** Alternative antigenic map conformation found upon comparing all 1000 optimizations (see Extended Data Fig. 4 and Supplementary Note 3). The map from optimization 568 is shown. **e,** The lowest stress antigenic map (optimization 1), with Procrustes arrows pointing towards the positions of each antigen and serum in the optimization 568 map conformation. **f,** Bayesian bootstrap blob size analysis. The antigen colour corresponds to the radius (AU) of a sphere of equal volume than each blob as displayed on the right. For interpretation, 1-2 AU differences correspond to the HI assay variation. Available via https://epiv-lab.github.io/H5-antigenically-central-vaccine/Supplementary_Data_3.html.

Supplementary Data 4 | The effect of removing single individual antigens and sera on the map geometry.

Each antigen and serum were individually removed from the antigenic map, and the full antigenic map was compared to the resulting maps, as detailed in Supplementary Note 3. The maps with the highest median Procrustes distance are displayed. **a-h,** Interactive versions of the antigenic map, represented as described for Supplementary Data 2. In each panel, the full antigenic map is displayed (117x29), and Procrustes arrows point at the positions of each antigen and serum in the map in which a single individual antigen (**a-c**) or serum (**d-h**) was removed, as indicated above each panel. The removed point is faded out and no Procrustes arrow is drawn. Ag.: Antigen; Sr.: Serum. Available via https://epiv-lab.github.io/H5-antigenically-central-vaccine/Supplementary_Data_4.html.

Supplementary Data 5 | A(H5) antigenic maps highlighting WHO candidate virus vaccines and antigens used in the ferret vaccination studies.

Interactive versions of the antigenic map (117x29), represented as described for Supplementary Data 2. Sera are not shown, and antigens of interest are highlighted as opaque spheres. **a,** Highlighting the WHO candidate virus vaccines (larger spheres) and the WHO CVV-like (smaller spheres) antigens (see Supplementary Table 2). **b,** Antigens used in the vaccination-challenge studies are highlighted as larger spheres. The antigenic maps can be rotated by clicking and dragging in the panel and scrolling allows zooming in and out. Available via https://epiv-lab.github.io/H5-antigenically-central-vaccine/Supplementary_Data_5.html.

Supplementary Data 6 | Mean antibody profiles upon vaccination with whole-inactivated vaccines containing mutated HA antigens.

An interactive version of the antibody profiles displayed in Fig. 2. For each HA vaccine antigen, the position, breadth and height of a mean serum per group are represented in the antigenic map from Fig. 1b. HA present in vaccine: **a,** $\text{Iraq}_{\text{VACC}}$ (n=2), **b,** $\text{CVA-Vietnam}_{\text{VACC}}$ (n=2), **c,** $\text{CVA-Indonesia}_{\text{VACC}}$ (n=2), and **d,** $\text{CVA-Anhui}_{\text{VACC}}$ (n=1). Representation is as described for Fig. 2. In addition, the map orientation can be changed by clicking and dragging within the visualization and scrolling allows zooming in and out. Antigen names can be visualized by hovering over the points. AU: antigenic unit; GMT: geometric mean titre. Available via https://epiv-lab.github.io/H5-antigenically-central-vaccine/Supplementary_Data_6.html.

Supplementary Data 7 | Individual antibody profiles upon vaccination with whole-inactivated vaccines containing mutated HA antigens.

Individual animal data used to generate mean antibody profiles displayed in Fig. 2 and Supplementary Data 6. For each HA vaccine antigen, the position, breadth and height of individual sera are represented in the antigenic map from Fig. 1b. HA present in vaccine: **a-b,** $Iraq_{VACC}$, **c-d,** CVA-$Vietnam_{VACC}$ and **e-f,** CVA-$Indonesia_{VACC}$. Using the same representation as Supplementary Data 6. Available via https://epiv-lab.github.io/H5-antigenically-central-vaccine/Supplementary_Data_7.html.

Supplementary Data 8 | Mean antibody profiles upon vaccination with split-inactivated vaccines containing wild-type HA antigens or the antigenically central HA antigen.

An interactive version of the antibody profiles displayed in Fig. 3. For each group, the position, breadth and height of a mean serum per group (n=6) are represented in the antigenic map from Supplementary Data 5b. **a-c,** Immune responses upon vaccination with A(H5N6) split-inactivated vaccines in the $H5N1_{Giza}$ challenge study or **d-f,** A(H5N1) split-inactivated vaccines in the $H5N6_{Sichuan}$ challenge study. HA antigens present in vaccine: **a** and **d,** $Anhui_{VACC}$, **b** and **e,** AC-$Anhui_{VACC}$, **c,** $Giza_{VACC}$ and **f,** $Sichuan_{VACC}$. Using the same representation as Supplementary Data 6. AU: antigenic unit; GMT: geometric mean titre. Available via https://epiv-lab.github.io/H5-antigenically-central-vaccine/Supplementary_Data_8.html.

Supplementary Data 9 | Individual antibody profiles of animals from the $H5N1_{Giza}$ vaccination-challenge study.

Individual immune responses upon vaccination with A(H5N6) split-inactivated vaccines in the $H5N1_{Giza}$ challenge study. Individual animal data used to generate mean antibody profiles displayed in Fig. 3 and Supplementary Data 8. For each HA vaccine antigen, the position, breadth and height of individual sera are represented in the antigenic map from Supplementary Data 5b. HA antigen present in vaccine: **a-f,** $Anhui_{VACC}$, **g-l,** AC-$Anhui_{VACC}$ and **m-r,** $Giza_{VACC}$. Using the same representation as Supplementary Data 6. Available via https://epiv-lab.github.io/H5-antigenically-central-vaccine/Supplementary_Data_9.html.

Supplementary Data 10 | Individual antibody profiles of animals from the $H5N6_{Sichuan}$ vaccination-challenge study.

Individual immune responses upon vaccination with A(H5N1) split-inactivated vaccines in the $H5N6_{Sichuan}$ challenge study. Individual animal data used to generate the mean antibody profiles displayed in Fig. 3 and Supplementary Data 8. For each HA vaccine antigen, the position, breadth and height of individual sera are represented in the antigenic map from Supplementary Data 5b. HA antigen present in vaccine: **a-f,** $Anhui_{VACC}$, **g-l,** AC-$Anhui_{VACC}$ and **m-r,** $Sichuan_{VACC}$. Using the same representation as Supplementary Data 6. Available via https://epiv-lab.github.io/H5-antigenically-central-vaccine/Supplementary_Data_10.html.

Supplementary Video Legend

Supplementary Video | A(H5) antigenic evolution over time.

The antigens and sera of the three-dimensional antigenic map constructed from the final 117x29 dataset (as shown in Fig. 1b and Supplementary Data 2) appear based on the year of isolation of the respective virus, as indicated in the top-right corner. Antigens are displayed as closed spheres and sera are displayed as open cubes. Antigens and sera are colour-coded based on the genetic HA clade, as indicated in the bottom-left legend. Each direction (x, y, z) represents antigenic distance, and one square of the grid corresponds to one antigenic unit, which is defined as a two-fold difference in HI titre. The antigenic map is oscillating for visualization purposes.
