# Supplementary material for: A vaccine central in A(H5) influenza antigenic space confers broad immunity
Source: Nature. 2025 Oct 15;647(8091):1005–13. doi: 10.1038/s41586-025-09626-3 (PMC12657240; doi:10.1038/s41586-025-09626-3)
Supplement: Supplementary file 2 — Reporting Summary [file 41586_2025_9626_MOESM2_ESM.pdf]

nature portfolio

Corresponding author(s): Mathilde Richard

Last updated by author(s): Aug 4, 2025

Reporting Summary

Nature Portfolio wishes to improve the reproducibility of the work that we publish. This form provides structure for consistency and transparency in reporting. For further information on Nature Portfolio policies, see our Editorial Policies and the Editorial Policy Checklist.

Statistics

For all statistical analyses, confirm that the following items are present in the figure legend, table legend, main text, or Methods section.

n/a	Confirmed	
☐	☒	The exact sample size (*n*) for each experimental group/condition, given as a discrete number and unit of measurement
☐	☒	A statement on whether measurements were taken from distinct samples or whether the same sample was measured repeatedly
☐	☒	The statistical test(s) used AND whether they are one- or two-sided<br>*Only common tests should be described solely by name; describe more complex techniques in the Methods section.*
☒	☐	A description of all covariates tested
☐	☒	A description of any assumptions or corrections, such as tests of normality and adjustment for multiple comparisons
☐	☒	A full description of the statistical parameters including central tendency (e.g. means) or other basic estimates (e.g. regression coefficient) AND variation (e.g. standard deviation) or associated estimates of uncertainty (e.g. confidence intervals)
☐	☒	For null hypothesis testing, the test statistic (e.g. *F*, *t*, *r*) with confidence intervals, effect sizes, degrees of freedom and *P* value noted<br>*Give P values as exact values whenever suitable.*
☒	☐	For Bayesian analysis, information on the choice of priors and Markov chain Monte Carlo settings
☒	☐	For hierarchical and complex designs, identification of the appropriate level for tests and full reporting of outcomes
☒	☐	Estimates of effect sizes (e.g. Cohen's *d*, Pearson's *r*), indicating how they were calculated

Our web collection on statistics for biologists contains articles on many of the points above.

Software and code

Policy information about availability of computer code

Data collection	Cell Olympus for micrographs acquisition.
Data analysis	R version 4.4.3. Python version 3.10.14. R package Racmacs, available at: https://acorg.github.io/Racmcas/ (version 1.2.3). Custom R and Python scripts were used, which are publicly available at https://github.com/epiv-lab/H5-antigenic-evolution (zenodo: https://doi.org/10.5281/zenodo.13237524) and https://github.com/epiv-lab/pepiniere (zenodo: https://doi.org/10.5281/zenodo.12751132). Python trimesh package (version 3.2.0). Python PyRacmacs package, available at https://github.com/iAvicenna/PyRacmacs. MAFFT version v7.515. IQ-Tree2 version 2.1.4_beta. ggtree version 1.4.11. LABEL (H5v2023 pre-release 1 (2023-05-05)). R Biostring package version 2.74.1. R rj3s package version 0.0.2. R ggplot2 package version 3.5.1. R flexdashboard package version 0.6.2. Webshot version 0.1.2.

For manuscripts utilizing custom algorithms or software that are central to the research but not yet described in published literature, software must be made available to editors and reviewers. We strongly encourage code deposition in a community repository (e.g. GitHub). See the Nature Portfolio guidelines for submitting code & software for further information.

Data

Policy information about availability of data

All manuscripts must include a data availability statement. This statement should provide the following information, where applicable:

- Accession codes, unique identifiers, or web links for publicly available datasets
- A description of any restrictions on data availability
- For clinical datasets or third party data, please ensure that the statement adheres to our policy

All data are available in the main text or the supplementary materials. Sequence accession numbers are available in Supplementary Table 1. Supplementary Data 1 to 10 are available via https://epiv-lab.github.io/H5-antigenically-central-vaccine/.

Research involving human participants, their data, or biological material

Policy information about studies with human participants or human data. See also policy information about sex, gender (identity/presentation), and sexual orientation and race, ethnicity and racism.

Reporting on sex and gender	Research does not involved human participants or human data.
Reporting on race, ethnicity, or other socially relevant groupings	Research does not involved human participants or human data.
Population characteristics	Research does not involved human participants or human data.
Recruitment	Research does not involved human participants or human data.
Ethics oversight	Research does not involved human participants or human data.

Note that full information on the approval of the study protocol must also be provided in the manuscript.

Field-specific reporting

Please select the one below that is the best fit for your research. If you are not sure, read the appropriate sections before making your selection.

☒ Life sciences ☐ Behavioural & social sciences ☐ Ecological, evolutionary & environmental sciences

For a reference copy of the document with all sections, see nature.com/documents/nr-reporting-summary-flat.pdf

Life sciences study design

All studies must disclose on these points even when the disclosure is negative.

Sample size	The use of 6 animals in vaccination challenge experiments makes it possible to demonstrate relevant differences between test groups (de Wit et al., J Virol, 2005, 79 (19): 12401) (Richardson, J Virol., 2005, 79 (2): 669-676) (Kreijtz et al, Vaccine, 2007). The current experimental set-up allows us to compare differences in viral replication between the vaccination groups. In addition, historical data concerning the difference and the spread between groups allows us to also estimate the group size:<br>n = 2'[{(z1-a / 2 + z1-b) s} / (m1-m0)] 2<br>a = 0.05<br>1-b = 0.8 (Power of 80)<br>s = 2.46 (spread between groups in log units)<br>m1 = 3.52 (difference between groups in log units)<br>m0 = 0<br><br>--> n=6
Data exclusions	No data were excluded from analyses
Replication	In vivo experiments were replicated 6 times (see above justification of a group size of 6 for vaccination-challenge experiments. A subset of the hemagglutination inhibition titrations were replicated twice or more to evaluate assay variation (presented in Ext. Figure 1A). Titrations of the ferret swabs and tissues were performed in quadriplicates.
Randomization	Animals were randomly allocated to the different groups. Randomization was not applicable to the other experiments.
Blinding	Animal experiments were not blinded to the investigators due to regulations that require knowledge of animal treatment for biosafety reasons. Pathological scoring and analyses were performed blindly. Titrations and hemagglutination inhibition assays were read out blindly.

Reporting for specific materials, systems and methods

We require information from authors about some types of materials, experimental systems and methods used in many studies. Here, indicate whether each material, system or method listed is relevant to your study. If you are not sure if a list item applies to your research, read the appropriate section before selecting a response.

Materials & experimental systems

n/a	Involved in the study
☐	☒ Antibodies
☐	☒ Eukaryotic cell lines
☒	☐ Palaeontology and archaeology
☐	☒ Animals and other organisms
☒	☐ Clinical data
☒	☐ Dual use research of concern
☒	☐ Plants

Methods

n/a	Involved in the study
☒	☐ ChIP-seq
☒	☐ Flow cytometry
☒	☐ MRI-based neuroimaging

Antibodies

Antibodies used	mouse IgG2a anti-influenza A nucleoprotein, H16-L10-4R5 (ATCC® HB-65™, RRID: CVCL_4524); mouse IgG2a isotype control (R&D, MAB003, RRID:AB_357345); goat anti-mouse IgG2a secondary antibody coupled to horseradish peroxidase (HRP) (Biorad, Star133A, RRID: AB_1102655P).
Validation	The mouse IgG2a anti-influenza A nucleoprotein was tested by testing several dilutions on a tissue with known positivity (ferret nasal turbinates and cat lung infected with pH1N1. The lowest dilution of antibody leading to a clear signal (red precipiate) was chosen. Specificity was assessed using an isotype control, for which the same amount of antibody as the primary antibody was used.

Eukaryotic cell lines

Policy information about cell lines and Sex and Gender in Research

Cell line source(s)	HEK293T and MDCK cells were acquired from ATCC
Authentication	None of the cell lines used were authenticated
Mycoplasma contamination	The cell banks were tested negative for mycoplasma.
Commonly misidentified lines (See ICLAC register)	None

Animals and other research organisms

Policy information about studies involving animals; ARRIVE guidelines recommended for reporting animal research, and Sex and Gender in Research

Laboratory animals	Mustela putorius furo, 6 to 1 year old
Wild animals	No wild animals were used in this study
Reporting on sex	males (sera production) and females (vaccination challenge experiment)
Field-collected samples	No field-collected samples were used
Ethics oversight	Ferret experiments were performed in strict compliance with the Dutch legislation on the protection of animals used for scientific purposes (2014, European Union directive 2010/63/EU implemented). Experiments were performed at the Erasmus Medical Center in Rotterdam, the Netherlands under a project license accredited by the Dutch competent authority (license number AVD101002015340). Study protocols were approved by the Erasmus Medical Center Animal Welfare Body (permit numbers 15-340-01, -04, -06, -22, -23, and -24).

Note that full information on the approval of the study protocol must also be provided in the manuscript.

Plants

Seed stocks	*Report on the source of all seed stocks or other plant material used. If applicable, state the seed stock centre and catalogue number. If plant specimens were collected from the field, describe the collection location, date and sampling procedures.*
Novel plant genotypes	*Describe the methods by which all novel plant genotypes were produced. This includes those generated by transgenic approaches, gene editing, chemical/radiation-based mutagenesis and hybridization. For transgenic lines, describe the transformation method, the number of independent lines analyzed and the generation upon which experiments were performed. For gene-edited lines, describe the editor used, the endogenous sequence targeted for editing, the targeting guide RNA sequence (if applicable) and how the editor was applied.*
Authentication	*Describe any authentication procedures for each seed stock used or novel genotype generated. Describe any experiments used to assess the effect of a mutation and, where applicable, how potential secondary effects (e.g. second site T-DNA insertions, mosiacism, off-target gene editing) were examined.*
