# Supplementary material for: A vaccine central in A(H5) influenza antigenic space confers broad immunity
Source: Nature. 2025 Oct 15;647(8091):1005–13. doi: 10.1038/s41586-025-09626-3 (PMC12657240; doi:10.1038/s41586-025-09626-3)
Supplement: Supplementary file 5 — Supplementary Data 1–10 [file 41586_2025_9626_MOESM5_ESM.zip › 2024-10-22817B-s5/Supplementary-Data-10.html]

Supplementary Data 10


Supplementary Data 10

## Row

### **a.** H5N6Sichuan challenge, AnhuiVACC, I

### **b.** H5N6Sichuan challenge, AnhuiVACC, II

### **c.** H5N6Sichuan challenge, AnhuiVACC, III

### **d.** H5N6Sichuan challenge, AnhuiVACC, IV

### **e.** H5N6Sichuan challenge, AnhuiVACC, V

### **f.** H5N6Sichuan challenge, AnhuiVACC, VI

## Row

### **g.** H5N6Sichuan challenge, AC-AnhuiVACC, I

### **h.** H5N6Sichuan challenge, AC-AnhuiVACC, II

### **i.** H5N6Sichuan challenge, AC-AnhuiVACC, III

### **j.** H5N6Sichuan challenge, AC-AnhuiVACC, IV

### **k.** H5N6Sichuan challenge, AC-AnhuiVACC, V

### **l.** H5N6Sichuan challenge, AC-AnhuiVACC, VI

## Row

### **m.** H5N6Sichuan challenge, SichuanVACC, I

### **n.** H5N6Sichuan challenge, SichuanVACC, II

### **o.** H5N6Sichuan challenge, SichuanVACC, III

### **p.** H5N6Sichuan challenge, SichuanVACC, IV

### **q.** H5N6Sichuan challenge, SichuanVACC, V

### **r.** H5N6Sichuan challenge, SichuanVACC, VI

## Row

**Supplementary Data 10 |** **Individual antibody
profiles of animals from the H5N6Sichuan**
**vaccination-challenge study.**   Individual immune
responses upon vaccination with A(H5N1) split-inactivated vaccines in
the H5N6Sichuan challenge study. Individual animal data used
to generate the mean antibody profiles displayed in Fig. 3 and
Supplementary Data 8. For each HA vaccine antigen, the position, breadth
and height of individual sera are represented in the antigenic map from
Supplementary Data 5b. HA antigen present in vaccine:
(**a**-**f**) AnhuiVACC,
(**g**-**l**) AC-AnhuiVACC and
(**m**-**r**) SichuanVACC. Using
the same representation as Supplementary Data 6.
