# Supplementary material for: A vaccine central in A(H5) influenza antigenic space confers broad immunity
Source: Nature. 2025 Oct 15;647(8091):1005–13. doi: 10.1038/s41586-025-09626-3 (PMC12657240; doi:10.1038/s41586-025-09626-3)
Supplement: Supplementary file 5 — Supplementary Data 1–10 [file 41586_2025_9626_MOESM5_ESM.zip › 2024-10-22817B-s5/Supplementary-Data-2.html]

Supplementary Data 2


Supplementary Data 2

## Row

####

####

## Row

## Row

**Supplementary Data 2 | Three-dimensional A(H5) influenza
antigenic map.**An interactive version of the three-dimensional antigenic map
constructed from the final 117x29 dataset, shown in Fig. 1b. Antigens
are displayed as closed spheres and sera are displayed as open cubes.
Antigens and sera are colour-coded based on the genetic HA clade, as
indicated on the right-hand side of the figure. Antigens and sera names
can be visualized by hovering over the points. Each direction (x, y, z)
represents antigenic distance and one square of the grid corresponds to
one antigenic unit, which is defined as a two-fold difference in HI
titre. The antigenic map can be rotated by clicking and dragging in the
panel. On the top right are different functions to explore the map and a
brief description of each function appears when hovering over. The total
map stress, mean stress per titre and mean stress per detectable titre
are indicated at the bottom left.
