# Supplementary material for: A vaccine central in A(H5) influenza antigenic space confers broad immunity
Source: Nature. 2025 Oct 15;647(8091):1005–13. doi: 10.1038/s41586-025-09626-3 (PMC12657240; doi:10.1038/s41586-025-09626-3)
Supplement: Supplementary file 5 — Supplementary Data 1–10 [file 41586_2025_9626_MOESM5_ESM.zip › 2024-10-22817B-s5/Supplementary-Data-3.html]

Supplementary Data 3


Supplementary Data 3

## Row

### **a.** Piecewise procrustes 3D to 4D, 1 piece

### **b.** Piecewise procrustes 3D to 4D, 2 pieces

### **c.** Triangulation blobs

## Row

### **d.** Alternative map conformation

### **e.** Comparison of map conformations

### **f.** Bootstrap volume radii

## Row

**Supplementary Data 3 | Validation of the A(H5) antigenic
map.**Interactive versions of the three-dimensional antigenic map,
represented as described for Supplementary Data 2. (**a**,
**b**) Piecewise Procrustes analysis (see Extended Data
Fig. 2 and detailed in Supplementary Note 3) comparing the antigenic
maps in three and four dimensions. The results of analysis with one (a)
and two (b) pieces are displayed. The antigen colour hue indicates which
piece it belongs to, and the shading indicates the Procrustes distance
according to the gradient displayed on the right, in antigenic units
(AU). (**c**) Triangulation blobs indicating the area in
which each datapoint can be located in the antigenic map without
increasing the total map stress by more than one unit.
(**d**) Alternative antigenic map conformation found upon
comparing all 1000 optimizations (see Extended Data Fig. 4 and
Supplementary Note 3). The map from optimization 568 is shown.
(**e**) The lowest stress antigenic map (optimization 1),
with Procrustes arrows pointing towards the positions of each antigen
and serum in the optimization 568 map conformation. (**f**)
Bayesian bootstrap blob size analysis. The antigen colour corresponds to
the radius (AU) of a sphere of equal volume than each blob as displayed
on the right. For interpretation, 1-2 AU differences correspond to the
HI assay variation.
