# Supplementary material for: A vaccine central in A(H5) influenza antigenic space confers broad immunity
Source: Nature. 2025 Oct 15;647(8091):1005–13. doi: 10.1038/s41586-025-09626-3 (PMC12657240; doi:10.1038/s41586-025-09626-3)
Supplement: Supplementary file 5 — Supplementary Data 1–10 [file 41586_2025_9626_MOESM5_ESM.zip › 2024-10-22817B-s5/Supplementary-Data-4.html]

Supplementary Data 4


Supplementary Data 4

## Row

### **a.** Ag: A/HONG-KONG/486/1997\_0

### **b.** Ag: A/TURKEY/65596/2006\_2-2

### **c.** Ag: A/GUANGZHOU/39715/2014\_2-3-4-4E

### **d.** Sr: A/HONGKONG/483B/1997

## Row

### **e.** Sr: A/GUANGZHOU/39715A/2014

### **f.** Sr: A/CHICKEN/JIANGSU/K0101B/2010

### **g.** Sr: A/DUCK/GIZA/15292SA/2015

### **h.** Sr: A/CHICKEN/VIETNAM/NCVD-15A59A/2015

## Row

**Supplementary Data 4 | The effect of removing single
individual antigens and sera on the map geometry.**Each antigen and serum were individually removed from the
antigenic map, and the full antigenic map was compared to the resulting
maps, as detailed in Supplementary Note 3. The maps with the highest
median Procrustes distance are displayed. (**a-h**)
Interactive versions of the antigenic map, represented as described for
Supplementary Data 2. In each panel, the full antigenic map is displayed
(117x29), and Procrustes arrows point at the positions of each antigen
and serum in the map in which a single individual antigen
(**a**-**c**) or serum
(**d**-**h**) was removed, as indicated above
each panel. The removed point is faded out and no Procrustes arrow is
drawn. Ag.: Antigen; Sr.: Serum.
