# Supplementary material for: A vaccine central in A(H5) influenza antigenic space confers broad immunity
Source: Nature. 2025 Oct 15;647(8091):1005–13. doi: 10.1038/s41586-025-09626-3 (PMC12657240; doi:10.1038/s41586-025-09626-3)
Supplement: Supplementary file 5 — Supplementary Data 1–10 [file 41586_2025_9626_MOESM5_ESM.zip › 2024-10-22817B-s5/Supplementary-Data-5.html]

Supplementary Data 5


Supplementary Data 5

## Row

### **a.**

### **b.**

## Row

**Supplementary Data 5 | A(H5) antigenic maps highlighting WHO
candidate virus vaccines and antigens used in the ferret vaccination
studies.**Interactive versions of the antigenic map (117x29), represented
as described for Supplementary Data 2. Sera are not shown and antigens
of interest are highlighted as opaque spheres. (**a**)
Highlighting the WHO candidate virus vaccines (larger spheres) and the
WHO CVV-like (smaller spheres) antigens (see Supplementary Table 2).
(**b**) Antigens used in the vaccination-challenge studies
are highlighted as larger spheres. The antigenic maps can be rotated by
clicking and dragging in the panel and scrolling allows zooming in and
out.
