# Supplementary material for: A vaccine central in A(H5) influenza antigenic space confers broad immunity
Source: Nature. 2025 Oct 15;647(8091):1005–13. doi: 10.1038/s41586-025-09626-3 (PMC12657240; doi:10.1038/s41586-025-09626-3)
Supplement: Supplementary file 5 — Supplementary Data 1–10 [file 41586_2025_9626_MOESM5_ESM.zip › 2024-10-22817B-s5/Supplementary-Data-6.html]

Supplementary Data 6


Supplementary Data 6

## Row

### **a.** IraqVACC

3.52 AU to center | GMT: 21 | 50 detectable titres

### **b.** CVA-VietnamVACC

2.70 AU to center | GMT: 32 | 78 detectable titres

## Row

### **c.** CVA-IndonesiaVACC

0.76 AU to center | GMT: 36 | 80 detectable titres

### **d.** CVA-AnhuiVACC

1.60 AU to center | GMT: 43 | 82 detectable titres

## Row

**Supplementary Data 6** | **Mean antibody
profiles upon vaccination with whole-inactivated vaccines containing
mutated HA antigens.**    
An interactive version of the antibody profiles displayed in Fig. 2. For
each HA vaccine antigen, the position, breadth and height of a mean
serum per group (n=2) are represented in the antigenic map from Fig. 1b.
HA present in vaccine: **a**, IraqVACC (n=2),
**b**, CVA-VietnamVACC (n=2),
**c**, CVA-IndonesiaVACC (n=2), and
**d**, CVA-AnhuiVACC (n=1). Representation is as
described for Fig. 2. In addition, the map orientation can be changed by
clicking and dragging within the visualization and scrolling allows
zooming in and out. Antigen names can be visualized by hovering over the
points. AU: antigenic unit; GMT: geometric mean titre.
