# Supplementary material for: A vaccine central in A(H5) influenza antigenic space confers broad immunity
Source: Nature. 2025 Oct 15;647(8091):1005–13. doi: 10.1038/s41586-025-09626-3 (PMC12657240; doi:10.1038/s41586-025-09626-3)
Supplement: Supplementary file 5 — Supplementary Data 1–10 [file 41586_2025_9626_MOESM5_ESM.zip › 2024-10-22817B-s5/Supplementary-Data-7.html]

Supplementary Data 7


Supplementary Data 7

## Row

### **a.** IraqVACC, I

### **b.** IraqVACC, II

### **c.** CVA-VietnamVACC, I

## Row

### **d.** CVA-VietnamVACC, II

### **e.** CVA-IndonesiaVACC, I

### F. CVA-IndonesiaVACC, II

## Row

**Supplementary Data 7 | Individual antibody profiles upon
vaccination with whole-inactivated vaccines containing mutated HA
antigens.**Individual animal data used to generate mean antibody profiles
displayed in Fig. 2 and Supplementary Data 6. For each HA vaccine
antigen, the position, breadth and height of individual sera are
represented in the antigenic map from Fig. 1b. HA present in vaccine:
(**a**, **b**) IraqVACC,
(**c**, **d**) CVA-VietnamVACC and
(**e**, **f**) CVA-IndonesiaVACC.
Using the same representation as Supplementary Data 6.
