# Supplementary material for: A vaccine central in A(H5) influenza antigenic space confers broad immunity
Source: Nature. 2025 Oct 15;647(8091):1005–13. doi: 10.1038/s41586-025-09626-3 (PMC12657240; doi:10.1038/s41586-025-09626-3)
Supplement: Supplementary file 5 — Supplementary Data 1–10 [file 41586_2025_9626_MOESM5_ESM.zip › 2024-10-22817B-s5/Supplementary-Data-8.html]

Supplementary Data 8


Supplementary Data 8

## Row

### **a.** H5N1Giza challenge, AnhuiVACC

3.51 AU to center | GMT: 9 | 27 detectable titres

### **b.** H5N1Giza challenge, AC-AnhuiVACC

2.25 AU to center | GMT: 27 | 69 detectable titres

### **c.** H5N1Giza challenge, GizaVACC

2.73 AU to center | GMT: 16 | 48 detectable titres

## Row

### **d.** H5N6Sichuan challenge, AnhuiVACC

3.58 AU to center | GMT: 6 | 7 detectable titres

### **e.** H5N6Sichuan challenge, AC-AnhuiVACC

1.70 AU to center | GMT: 10 | 38 detectable titres

### **f.** H5N6Sichuan challenge, SichuanVACC

4.23 AU to center | GMT: 7 | 21 detectable titres

## Row

**Supplementary Data 8 | Mean antibody profiles upon
vaccination with split-inactivated vaccines containing wild-type HA
antigens or the antigenically central HA antigen.**An interactive version of the antibody profiles displayed in
Fig. 3. For each group, the position, breadth and height of a mean serum
per group (n=6) are represented in the antigenic map from Supplementary
Data 5b. (**a**-**c**) Immune responses upon
vaccination with A(H5N6) split-inactivated vaccines in the
H5N1Giza challenge study or
(**d**-**f**) A(H5N1) split-inactivated
vaccines in the H5N6Sichuan challenge study. HA antigens
present in vaccine: (**a, d**) AnhuiVACC,
(**b, e)** AC-AnhuiVACC, (**c**)
GizaVACC and (**f**) SichuanVACC.
Using the same representation as Supplementary Data 6. AU: antigenic
unit; GMT: geometric mean titre.
