# Supplementary material for: A vaccine central in A(H5) influenza antigenic space confers broad immunity
Source: Nature. 2025 Oct 15;647(8091):1005–13. doi: 10.1038/s41586-025-09626-3 (PMC12657240; doi:10.1038/s41586-025-09626-3)
Supplement: Supplementary file 5 — Supplementary Data 1–10 [file 41586_2025_9626_MOESM5_ESM.zip › 2024-10-22817B-s5/Supplementary-Data-9.html]

Supplementary Data 9


Supplementary Data 9

## Row

### **a.** H5N1Giza challenge, AnhuiVACC, I

### **b**. H5N1Giza challenge, AnhuiVACC, II

### **c.** H5N1Giza challenge, AnhuiVACC, III

### **d.** H5N1Giza challenge, AnhuiVACC, IV

### **e.** H5N1Giza challenge, AnhuiVACC, V

### **f.** H5N1Giza challenge, AnhuiVACC, VI

## Row

### **g.** H5N1Giza challenge, AC-AnhuiVACC, I

### **h.** H5N1Giza challenge, AC-AnhuiVACC, II

### **i.** H5N1Giza challenge, AC-AnhuiVACC, III

### **j.** H5N1Giza challenge, AC-AnhuiVACC, IV

### **k.** H5N1Giza challenge, AC-AnhuiVACC, V

### **l.** H5N1Giza challenge, AC-AnhuiVACC, VI

## Row

### **m.** H5N1Giza challenge, GizaVACC, I

### **n.** H5N1Giza challenge, GizaVACC, II

### **o.** H5N1Giza challenge, GizaVACC, III

### **p.** H5N1Giza challenge, GizaVACC, IV

### **q.** H5N1Giza challenge, GizaVACC, V

### **r.** H5N1Giza challenge, GizaVACC, VI

## Row

**Supplementary Data 9 | Individual antibody profiles of
animals from the H5N1Giza vaccination-challenge
study.**Individual immune responses upon vaccination with A(H5N6)
split-inactivated vaccines in the H5N1Giza challenge study.
Individual animal data used to generate mean antibody profiles displayed
in Fig. 3 and Supplementary Data 8. For each HA vaccine antigen, the
position, breadth and height of individual sera are represented in the
antigenic map from Supplementary Data 5b. HA antigen present in vaccine:
(**a**-**f**) AnhuiVACC,
(**g**-**l**) AC-AnhuiVACC and
(**m**-**r**) GizaVACC. Using the
same representation as Supplementary Data 6.
